# Predicting the severity of white matter lesions among patients with cerebrovascular risk factors based on retinal images and clinical laboratory data: a deep learning study

**DOI:** 10.3389/fneur.2023.1168836

**Published:** 2023-07-10

**Authors:** Liming Shu, Kaiyi Zhong, Nanya Chen, Wenxin Gu, Wenjing Shang, Jiahui Liang, Jiangtao Ren, Hua Hong

**Affiliations:** ^1^Department of Neurology, Seventh Affiliated Hospital, Sun Yat-sen University, Shenzhen, China; ^2^Department of Neurology, Second Affiliated Hospital of Guangzhou Medical University, Guangzhou, China; ^3^Department of Neurology, First Affiliated Hospital, Sun Yat-sen University, Guangzhou, China; ^4^School of Data and Computer Science, Sun Yat-sen University, Guangzhou, China; ^5^Department of Medical Imaging, Sun Yat-sen University Cancer Center, Guangzhou, China; ^6^Guangdong Key Laboratory of Non-human Primate Research, Guangdong-Hongkong-Macau Institute of CNS Regeneration, Jinan University, Guangzhou, China

**Keywords:** cerebrovascular disease, white matter lesions, retinal images, deep learning, prediction model

## Abstract

**Background and purpose:**

As one common feature of cerebral small vascular disease (cSVD), white matter lesions (WMLs) could lead to reduction in brain function. Using a convenient, cheap, and non-intrusive method to detect WMLs could substantially benefit to patient management in the community screening, especially in the settings of availability or contraindication of magnetic resonance imaging (MRI). Therefore, this study aimed to develop a useful model to incorporate clinical laboratory data and retinal images using deep learning models to predict the severity of WMLs.

**Methods:**

Two hundred fifty-nine patients with any kind of neurological diseases were enrolled in our study. Demographic data, retinal images, MRI, and laboratory data were collected for the patients. The patients were assigned to the absent/mild and moderate–severe WMLs groups according to Fazekas scoring system. Retinal images were acquired by fundus photography. A ResNet deep learning framework was used to analyze the retinal images. A clinical-laboratory signature was generated from laboratory data. Two prediction models, a combined model including demographic data, the clinical-laboratory signature, and the retinal images and a clinical model including only demographic data and the clinical-laboratory signature, were developed to predict the severity of WMLs.

**Results:**

Approximately one-quarter of the patients (25.6%) had moderate–severe WMLs. The left and right retinal images predicted moderate–severe WMLs with area under the curves (AUCs) of 0.73 and 0.94. The clinical-laboratory signature predicted moderate–severe WMLs with an AUC of 0.73. The combined model showed good performance in predicting moderate–severe WMLs with an AUC of 0.95, while the clinical model predicted moderate–severe WMLs with an AUC of 0.78.

**Conclusion:**

Combined with retinal images from conventional fundus photography and clinical laboratory data are reliable and convenient approach to predict the severity of WMLs and are helpful for the management and follow-up of WMLs patients.

## 1. Introduction

Stroke is a leading cause of mortality and long-term disability, especially in low- and middle-income countries (1). In China, among 28.76 million prevalent cases of stroke in 2019, and most of the cases were ischemic stroke (2). Cerebral small vascular disease (cSVD), including white matter lesions (WMLs), is a major challenge to brain health, accounting for approximately 30% of the cause of ischemic stroke (3). It was estimated that 36% of cSVD cases were due to WMLs (4).

The severity of WMLs is negatively associated with cognitive function, especially in the patients of Fazekas 3 and 4 WMLs (5, 6). In addition, WMLs was also association with new-onset depression in older people in the community and the risk of post-stroke depression and dysfunction of bowel and bladder (7). WMLs are associated with poorer gait and balance (8). And WMLs are a strong predictor of the incident stroke (9). The severity of WMLs progressed over time. The Leukoaraiosis and Disability Study showed that about 73.6% of the participants had WMLs progression during 3 years follow-up and among the patients with severe WMLs, more than 80% of them had WMLs progression (10). Thus, regular screening is crucial for the early identification of WMLs and the prevention of its progression. Magnetic resonance imaging (MRI) has been widely used to detect WMLs *in vivo* (11). However, MRI is not available for all the candidates who needed WMLs screening or diagnosis due to insufficient economic resource, unavailability of MRI equipment, contraindication of MRI and so on. Convenient substitution of MRI to assess WMLs is helpful for patient management and economic efficiency.

It’s worth noting that the brain and retinal vasculature are homology (12). More specifically, the blood–retinal barrier mirrors the blood–brain barrier. In addition, the microcirculation of the brain and retina share a similar autoregulation function to maintain blood flow (13). The retinal microcirculation has a similar embryonic, anatomical, and physiological basis to that of small intracranial vessels (14, 15). Thus, the retina is a window through which vasculature and neural tissues can be dynamically and non-invasively observed. Retinal blood vessel lesions have been found to be independently associated with WMLs (16–18).

Significant progress in deep learning classifiers has been achieved in medical imaging fields, such as radiology, dermatology, pathology and ophthalmology (19). Among various deep learning techniques, convolutional neural networks make automatic, efficient and accurate image-based diagnosis possible (20, 21). Specifically, deep learning models based on retinal images rival ophthalmologists when screening diabetic retinopathy (22). Moreover, there is early evidence that identification of central nervous system diseases based on retinal images is possible. Recently, texture characteristics of retinal images discovered by automated retinal image analysis have been found to predict WMLs in community participants (23). A deep learning algorithm has been applied to raw retinal images was put forward to predict WMLs (24). However, it is worth noticing that the capability to predict WMLs of these retinal images-based models needs further improvement. Thus, a comprehensive model that not only includes retinal images but also these clinical data need to be developed. A recent study developed a comprehensive model combined a deep learning model based on retinal images and clinical information, which accurately predicted chronic kidney disease and type 2 diabetes (25). Concerning that hypertension (26), hyperlipidemia (27), impaired glucose metabolism (28), kidney failure (29) and systemic inflammation (30) are risk factors of WMLs, incorporating the clinical information of these risk factors into the retinal images-based prediction model may improve the capability of prediction and enhance the clinical practicability of the model. To the best of our knowledge, no studies have been conducted to develop a deep learning model using retinal images combined with the clinical laboratory data to predict the severity of WMLs.

Therefore, based on the previous findings that retina and its vasculature share similarity with cerebral small vessels, we aimed to develop a comprehensive model including retinal images and clinical laboratory data to predict WMLs severity, which may facilitate screening WMLs patients and follow-up.

We first built up a ResNet deep learning neural network to analyze correlation between the retinal images and the severity of WMLs. Then, we fused a clinical-laboratory signature based on the clinical laboratory data. A model combining demographic-clinical characteristics, the clinical-laboratory signature, and the outputs of the retina deep learning neural network was further developed to predict the severity of WMLs. To test the significance of the retinal images on predicting the severity of WMLs, the clinical model only included demographic-clinical characteristics and the clinical-laboratory signature. The performance of predicting WMLs severity of the two models was compared.

## 2. Methods

### 2.1. Patient selection

The clinical documentations of patients admitted to the Department of Neurology, the First Affiliated Hospital of Sun Yat-sen University, from January 2018 to January 2020 were retrospectively reviewed. The inclusion criteria were as follows: (1) patients who underwent nonmydriatic fundus photography; (2) patients who underwent brain MRI; and (3) over 18 years old. The exclusion criteria were as follows: (1) poor quality nonmydriatic fundus photography or MRI images; (2) missing clinical data; (3) WMLs caused by hereditary diseases, immune disorder, or infection (e.g., Fabry disease, cerebral autosomal dominant arteriopathy with subcortical infarcts and leukoencephalopathy, cerebral autosomal recessive arteriopathy with subcortical infarcts and leukoencephalopathy, multiple sclerosis, neuromyelitis optica spectrum disorder, encephalitis); (4) intracranial hypertension or hydrocephalus; (5) major brain edema or intracerebral hemorrhage; (6) primary ophthalmological diseases impairing the retina or hindering observation of the retina by fundus photography; (7) recent (<6 months) ophthalmological surgery; and (8) concomitant significant systemic infection. Demographic-clinical data, including age, sex, systolic blood pressure (SBP) and diastolic blood pressure (DBP) on admission, were collected. Comorbidities, including hypertension, diabetes mellitus, statin usage, atrial fibrillation, coronary heart disease, previous stroke or transient ischemic attack (TIA), were documented. Hypertension was defined as systolic blood pressure of ≥140 mmHg or diastolic blood pressure of ≥90 mmHg or current treatment with anti-hypertensive medications. Diabetes mellitus was defined as fasting plasma glucose ≥7.0 mmol/L or hemoglobin A1c (HbA1c) ≥ 6.5% or current treatment with blood glucose lowering medication. This study met the criteria of the Declaration of Helsinki, and it was approved by the Ethics Committee of the First Affiliated Hospital of Sun Yat-sen University, which waived the requirement for written informed consent due to this study was retrospectively designed [No. (2022) 202].

### 2.2. Magnetic resonance imaging acquisition and analysis

All patients underwent brain MRI at 3 Tesla scanners (Siemens Trio Tim or Siemens Verio). In the present study, T1-weighted imaging (T1WI), T2-weighted imaging (T2WI), T2-fluid attenuation inversion recovery (T2-FLAIR), and diffusion-weighted imaging (DWI) were collected for all patients. The parameters were as follows: (1) T1WI, repetition time (TR)/echo time (TE) 500/8.9 ms, and slice thickness (SL) 6.0 mm; (2) T2WI, TR/TE = 4000/100 ms, and SL 6.0 mm; (3) T2-FLAIR, TR/TE = 9000/111 ms, inversion recovery 2,500 ms, and SL 6.0 mm; and (4) TR/TE = 5800/100 ms, b = 0/1000 s/mm2, and SL 5.0 mm.

**Figure 1:**
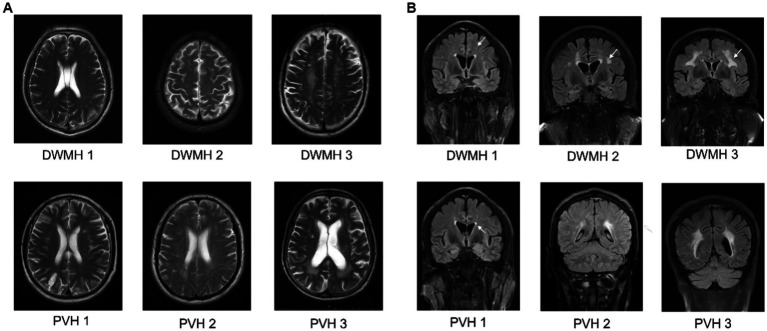
The transverse **(A)** and coronal **(B)** sections of MRI of Fazekas scale to semi-quantitatively quantify the WMLs severity. For deep white matter hyperintensity (DWMH): punctate foci, score 1; small confluences, score 2; large confluences, score 3. For periventricular hyperintensity (PVH): caps or pencil-thin linings, score 1; smooth halos with diameters of 6 mm ~ 10 mm, score 2; irregular lesions with diameters of more than 10 mm, score 3. DWMH, deep white matter hyperintensity; PVH, periventricular hyperintensity.

WMLs were analyzed on T2WI and T2-FLAIR images and semi-quantitatively graded based on the Fazekas grading system. Periventricular hyperintensity (PVH) was scored as 0 for the absence of any lesions, 1 for cap or pencil-like lesions, 2 for halo-like lesions with diameters of 6 mm ~ 10 mm, and 3 for irregular lesions with diameters of more than 10 mm. Deep white matter hyperintensity (DWMH) was scored as 0 for the absence of any lesion, 1 for punctate-like lesions, 2 for small confluent lesions, and 3 for large confluent lesions (Figure 1). For patients with significant brain edema induced by ischemic stroke or intracerebral hemorrhage, the Fazekas score was defined by the presence of a WML in the contralateral hemisphere. The scores of PVH and DWMH were further combined to represent the overall WMLs severity (31). The summation of the PVH score and DWMH score is the final Fazekas score that ranged from 0 to 6. The final Fazekas score was determined by two vascular neurologists (LS and KZ), and any discordant results between the two observers were resolved by consensus. Thus, the numbers of the patients of each final Fazekas score were as follow: final Fazekas score 0 (*n* = 81), final Fazekas score 1 (*n* = 62), final Fazekas score 2 (*n* = 51), final Fazekas score 3 (*n* = 29), final Fazekas score 4 (*n* = 16), final Fazekas score 5 (*n* = 8), and final Fazekas score 6 (*n* = 12). To minimize the redundancy and maximize the clinical practicability of our model, we assigned the patients with final Fazekas score 0 to 2 in the absent/mild WMLs group and the patients with final Fazekas score 3 to 6 in the moderate–severe WMLs group.

### 2.3. Retinal images acquisition

**Figure 2:**
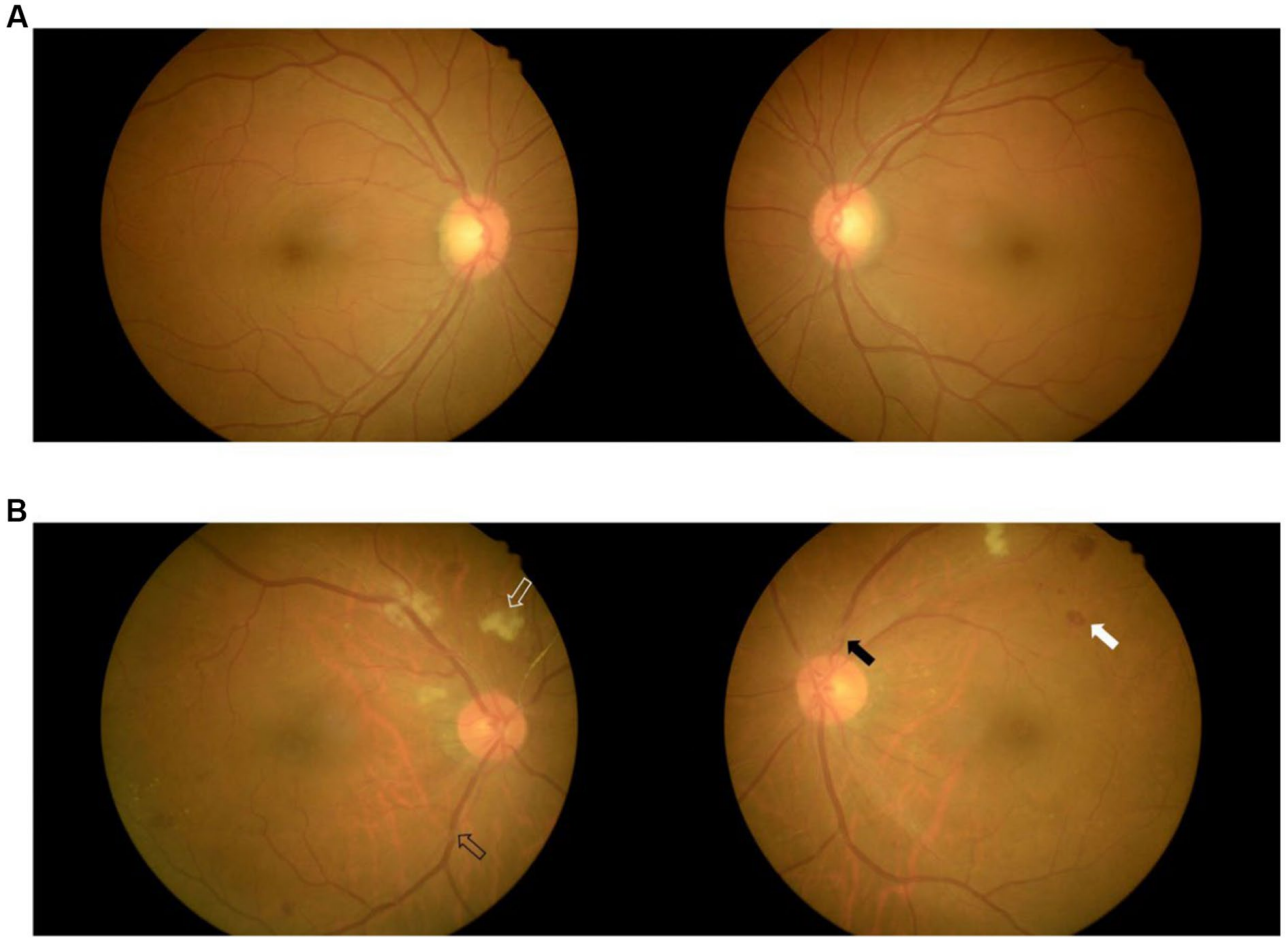
Fundus images of two patients with mild WMLs **(A)** and severe WMLs **(B)**. The white hollow arrow represents rigid exudation, the black hollow arrow represents arteriovenous pressure, the white arrow represents microhemorrhage, and the black arrow represents retinal artery stenosis. WMLs, white matter lesions.

Fundus photography was performed on both eyes for all patients without pupil dilatation (at a KOWA nonmyd7, Japan). In brief, patients sat in front of the fundus photography system and were instructed to focus on the center of the viewing system while the fundus was examined. The field of view was set to 45° to enable visualization of the posterior portion of the examined eye. The position of the camera and focal length were finely adjusted so that the optic disk and macula were clearly viewed. The images of both eyes were stored in a workstation for further analysis (Figure 2).

### 2.4. Deep learning neural network for retinal images

We used ResNet-18 model as the training model to predict the present moderate–severe WMLs in brain MRI scan by using retinal images. ResNet-18 is a residual network pretrained using the ImageNet dataset, but the associated fully connected softmax layer has been replaced by a supervised classifier. The pre-trained model used in our study is available on the code repository.^1^ We further modified this pre-trained model for our study. The convolution kernel of the convolutional layer is 7 × 7. The max pooling layer was directly performed by a convolutional layer with stride 2, followed by batch normalization after each convolution operation and before the ReLU activation function. The image was resized to 224 × 224 to match the input image size required by ResNet-18. Then we used a linear classifier to perform feature scaling on the 2048-dimensional output feature vector of the last convolutional layer for dimensionality reduction, and set the final output layer dimension as 2. Each network includes three main modules: the input module, the output module, and the intermediate convolution module. We used the cross-entropy loss function as objective function. The optimizer was Adam, learning rate was 0.001. Finally, the deep-learning model produced a single value ranged 0 to 1 for each retinal image. This value indicates the probability of moderate/severe WMLs that was predicted by this deep-learning model and we took this value for further analysis. The class activation maps for each retina were generated to visualize the weight of predicting WMLs in the sub-regions of retina. The class activation maps for each retinal images were generated by the Grad-CAM algorithm.

### 2.5. Clinical-laboratory signature

Clinical-laboratory data were examined for all patients. The clinical-laboratory data can be concisely divided into four regiments, including lipid-glucose metabolism, systemic inflammation, and liver and renal function. In detail, the lipid-glucose metabolism regimen included total cholesterol (CHOL), total triglyceride (TG), low density lipoprotein cholesterol (LDL-c), high density lipoprotein cholesterol (HDL-c), apolipoprotein-E (Apo-E), apolipoprotein-A1 (Apo-A1), apolipoprotein-B (Apo-B), Apo-A1/Apo-B ratio, lipoprotein-α (Lp-α), fasting blood glucose (FBG), HbA1c, free fat acid (FFA), fructosamine, and homocysteine. The systemic inflammation regimen included C-reactive protein (CRP), high-sensitivity C-reactive protein (Hs-CRP), and the Hs-CRP/CRP ratio. The liver function regimen included aspartate aminotransferase (AST), alanine aminotransferase (ALT), total protein (TP), total bilirubin (TBIL), albumin, globulin, and albumin/globulin ratio (A/G ratio). The renal function regimen, including creatine and uric acid (UA).

We developed a clinical-laboratory signature representing the metabolism, systemic inflammation, liver and renal function. A least absolute shrinkage and selection operator (LASSO) logistic regression model was implemented to eliminate collinearity, reduce overfitting, filter and select the clinical laboratory data that correlate with moderate–severe WMLs by tuning the hyperparameters that minimize Akaike information criteria (AIC).

### 2.6. Development of the WMLs severity prediction model

We developed a combined model including demographic-clinical characteristics, the clinical-laboratory signature, and the outputs of the retina deep learning neural network to predict WMLs severity. The demographic-clinical characteristics used in the predictive model were age, sex, SBP and DBP on admission. Age was included in the model as a continuous variable. Male was coded as 1 and female was coded as 0. SBP was further transformed into a hierarchical variable by defining values <140 mmHg as 0, values of 140 ~ 159 mmHg as 1, values of 160 ~ 179 mmHg as 2, and values of ≥180 mmHg as 3. Similarly, DBP was transformed by defining values <90 mmHg as 0, values of 90 ~ 99 mmHg as 1, values of 100 ~ 109 mmHg as 2, and values ≥110 mmHg as 3. The combined model was formulated based on the results of multivariate logistic regression that minimized the penalty parameter conducted by 10-fold cross-validation. Furthermore, a clinical model including demographic-clinical characteristics and clinical-laboratory signatures was generated based on the results of multivariate logistic regression that minimized the penalty parameter conducted by 10-fold cross-validation without the outputs of retina deep learning neural network.

### 2.7. Statistics

Data for continuous variables are summarized as the mean and standard deviation. Categorical variables were summarized by number and percentage. Univariate logistic regression was carried out in R (v4.0.5) to explore the relationship among demographic-clinical characteristics, individual clinical laboratory data and WMLs severity. The continuous variables were compared by independent-sample t test between patients in the absent/mild WMLs group and patients in the moderate–severe WMLs group. The categorical variables were compared by the chi-square test or Fisher’s exact test. Univariate logistic regression was used to explore the association between clinical-laboratory indices and dichotomized WMLs severity. Odds ratios (ORs) and 95% confident intervals (95% CIs) were used to predict the association between clinical-laboratory data and WMLs severity. The predictive model development and compilation of the clinical-laboratory signature were conducted in Python (v 3.7) and the scikit-learn package.^2^ The performance of the clinical-laboratory signature, the retinal images, the combined model, and the clinical model was comprehensively predicted by the receiver operating characteristic curve. The area under curve (AUC), accuracy, precision, recall, F1 score, sensitivity, and specificity were calculated. The coefficient of determination (*R*^2^) and recall rate were further utilized to predict the performance of the clinical-laboratory signature, and the clinical mode.

## 3. Results

### 3.1. Patient clinical characteristics

Two hundred fifty-nine patients were enrolled in our study. The mean age of all patients was 52.3 ± 13.5 years. More than half of the included patients were male. The mean SBP of all patients was 140.4 ± 24.8 mmHg. The mean DBP of all patients was 87.7 ± 15.4 mmHg. The most common comorbidity was hypertension, which was present in 56.8% of all patients. Current smoking (35.1%), statin usage (21.6%), diabetes mellitus (17.4%), alcohol abuse (14.7%) and previous ischemic stroke/TIA (18.5%) were also common among all patients. A small proportion of the patients had atrial fibrillation (2.3%) and coronary heart disease (3.5%). Furthermore, the clinical characteristics of the patients with absent/mild and moderate–severe WMLs were compared. The patients with moderate–severe WMLs were significantly older than those with absent/mild WMLs (*p* < 0.001). The SBP and DBP on admission were higher in the patients with moderate–severe WMLs than in those with absent/mild WMLs (*p* < 0.001). The proportions of hypertension, previous ischemic stroke/TIA, and atrial fibrillation were higher in the patients with moderate–severe WMLs than in those with absent/mild WMLs, while the frequency of diabetes mellitus was higher in the patients with absent/mild WMLs. The clinical characteristics of all patients are summarized in Table 1. The diagnoses of our patients of each WMLs group were demonstrated in the Supplementary Table S2. The majority of our patients were diagnosed with cerebral infarction (65.8% for the mild/absent WMLs group, 75.8% for the moderate–severe WMLs group). The proportions of cerebral infarction were not significantly different between the mild/absent and moderate–severe WMLs groups (*p* = 0.127; Supplementary Table S2). The number of other diagnoses was very small. Hence, the results of the chi-square test for these diagnoses were not reliable. To avoid mistranslating the results, we did not present the *p* values of these diagnoses.

**Table 1:** Clinical characteristics of all patients.

Clinical Characteristics	All patients (*n* = 259)	Absent/mild WMLs (*n* = 193)	Moderate–severe WMLs (*n* = 66)	*p* value
Age (y, M ± SD)	52.3 ± 13.5	49.5 ± 13.2	60.5 ± 10.6	<0.001
Sex, male n (%)	175 (67.6%)	129 (66.8)	46 (69.7)	0.78
Systolic blood pressure (mmHg, M ± SD)	140.4 ± 24.8	135.7 ± 23.6	154.4 ± 23.3	<0.001
Diastolic blood pressure (mmHg, M ± SD)	87.7 ± 15.4	85.2 ± 14.8	95.1 ± 14.4	<0.001
*Comorbidities, n (%)*				
Hypertension	147 (56.8%)	95 (49.2)	52 (78.8)	<0.001
Diabetes mellitus	45 (17.4%)	40 (20.7)	5 (7.6)	0.02
Statin usage	56 (21.6%)	37 (19.2)	19 (28.8)	0.14
Previous ischemic stroke/TIA	48 (18.5%)	28 (14.5)	20 (30.3)	0.008
Atrial fibrillation	6 (2.3%)	2 (1.0)	4 (6.1)	0.04
Coronary heart disease	9 (3.5%)	7 (3.6)	2 (3.0)	1.00
Current smoking	91 (35.1%)	66 (34.2)	25 (37.9)	0.70
Alcohol abuse	38 (14.7%)	31 (16.1)	7 (10.6)	0.38

y, year; *n*, number; WMLs, white matter lesions; TIA, transient ischemic attack; M, mean; SD, standard deviation.

### 3.2. The association between clinical-laboratory data and WMLs severity

The association between clinical-laboratory Data and WMLs severity was summarized by univariate logistic regression (Table 2). Homocysteine (OR: 1.05, 95%CI: 1.01–1.08), globulin (OR: 1.11, 95%CI: 1.04–1.17), UA (OR: 1.00, 95%CI: 1.00–1.01), and creatine (OR: 1.03, 95%CI: 1.02–1.04) were positively associated with moderate–severe WMLs. Meanwhile, a decreased A/G ratio was associated with moderate–severe WMLs. Furthermore, LASSO regression was used to select the clinical-laboratory indices. Globulin (*β* = 0.01157, *p* < 0.05), creatine (*β* = 0.00407; *p* < 0.05), ApoA1 (*β* = 0.08155; *p* < 0.05), and TG (*β* = −0.00494; *p* < 0.05) were included in the regression model to fuse a clinical-laboratory signature for each patient.

**Table 2:** Univariate logistics regression of clinical-laboratory indices and WMLs severity.

Clinical-laboratory Indices	OR (95%CI)	*p* value
*Metabolism*		
Hemoglobin A1c	0.85 (0.65–1.10)	0.22
Fasting blood glucose	1.04 (0.93–1.16)	0.50
Fructosamine	0.61 (0.16–2.42)	0.49
Total cholesterol	1.18 (0.94–1.46)	0.15
Total triglyceride	0.85 (0.64–1.14)	0.28
Low density lipoprotein cholesterol	1.29 (0.94–1.77)	0.12
High density lipoprotein cholesterol	1.58 (0.59–4.25)	0.36
Apolipoprotein-A1	1.76 (0.52–5.90)	0.36
Apolipoprotein-B	2.58 (0.84–7.90)	0.10
Apolipoprotein-A1/Apolipoprotein-B ratio	0.76 (0.42–1.38)	0.37
Apolipoprotein-E	1.00 (0.98–1.02)	0.79
Lipoprotein-α	1.00 (1.00–1.00)	0.98
Free fat acid	1.00 (1.00–1.00)	0.52
Homocysteine	1.05 (1.01–1.08)	0.01
*Liver function*		
Aspartate aminotransferase	1.01 (0.98–1.04)	0.38
Alanine aminotransferase	1.00 (0.98–1.02)	0.98
Total protein	1.00 (1.00–1.00)	0.68
Albumin	0.98 (0.91–1.06)	0.64
Globulin	1.11 (1.04–1.17)	<0.001
Albumin/globulin ratio	0.23 (0.09–0.60)	<0.001
Total bilirubin	1.03 (0.97–1.08)	0.37
*Renal function*		
Uric acid	1.00 (1.00–1.01)	0.04
Creatine	1.03 (1.02–1.04)	<0.001
*Systemic inflammatory indices*		
C-reactive protein	1.00 (0.98–1.02)	0.84
High-sensitivity C-reactive protein	1.00 (0.99–1.02)	0.73
High-sensitivity C-reactive protein / C-reactive protein ratio	0.92 (0.79–1.08)	0.29

WMLs, white matter lesions; OR, odds ratio; CI, confidence interval.

### 3.3. Prediction of severity of WMLs

#### 3.3.1. Performance of retinal deep learning neural network

**Figure 3:**
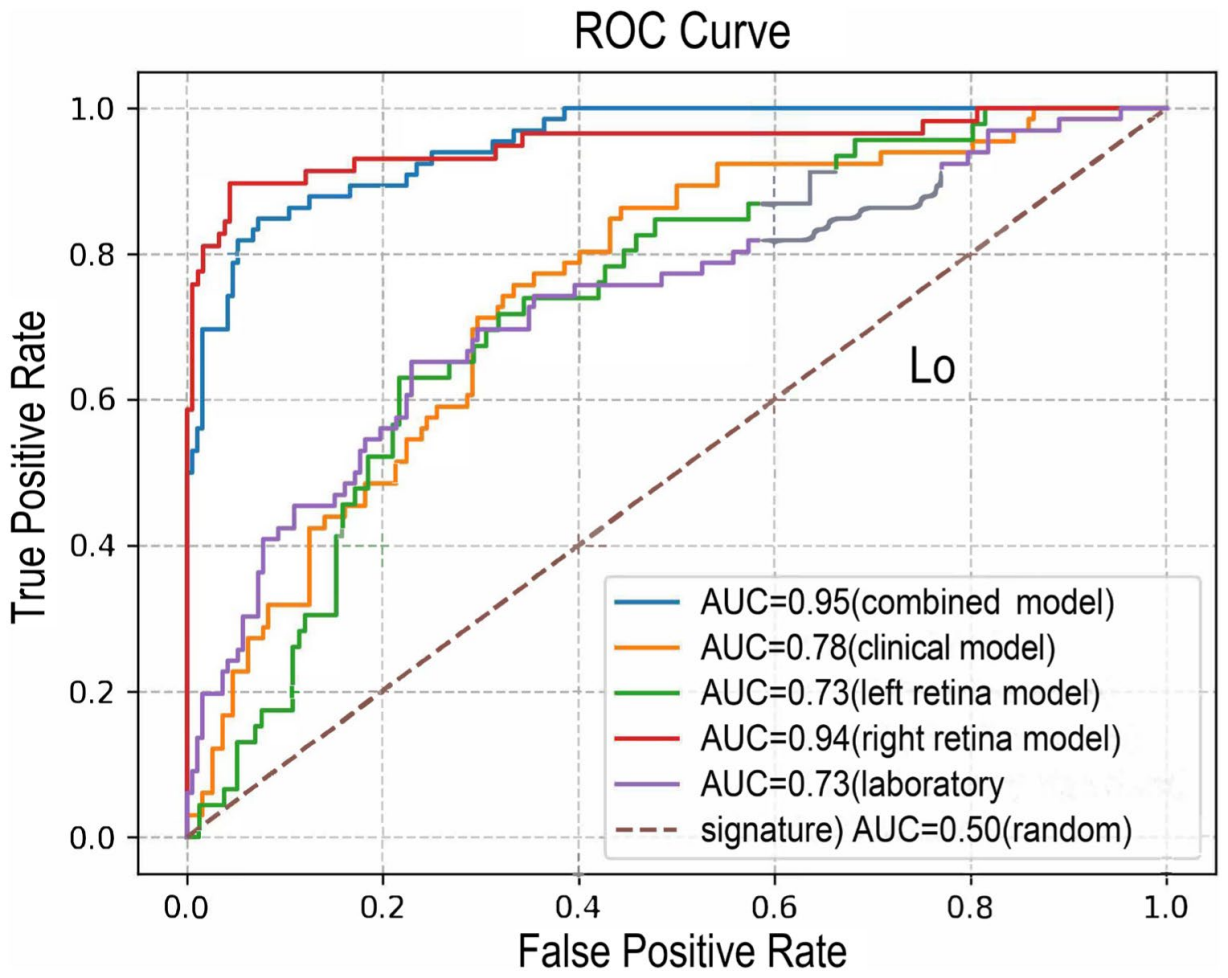
The ROC curves of the combined model (model 1, AUC = 0.95), the clinical model (model 2, AUC = 0.78), left retina model (AUC = 0.73), right retina model (AUC = 0.94) and laboratory signature model (AUC = 0.73). ROC, receiver operating characteristic; AUC, area under the ROC curve.

In this study, 204 images of retinas from the left eyes and 240 images from right eyes from total 259 participants were supplied to two ResNet-18 deep learning neural network frameworks to predict the dichotomized WML severity. The class activation maps and architecture was presented in the Supplementary Figure S1, S2. The models based on both sides of the retina could predict moderate–severe WMLs. The AUC, accuracy, precision, recall, F1 score, sensitivity, and specificity of left retina model predicting moderate–severe WMLs on the test set were 0.73, 0.75, 0.38, 0.13, 0.19, 0.13, and 0.94, respectively (Figure 3; Supplementary Table S1). The model based on the right retina was more powerful to predict WMLs severity. The model based on the right retina predicted moderate–severe WMLs with an AUC of 0.94, accuracy of 0.93, precision of 0.87, recall rate of 0.83, F1 score of 0.85, sensitivity of 0.83, specificity of 0.96 (Figure 3; Supplementary Table S1). The class activation maps indicated high weight of predicting moderate–severe WMLs in the regions containing retinal vasculature (Supplementary Figure S1).

#### 3.3.2. The laboratory signature and the clinical model

This clinical-laboratory signature included globulin, creatine, Apo-A1, and TG to predict moderate–severe WMLs with a determination *R*^2^ of 0.14. This signature showed an AUC of 0.73, accuracy of 0.77, precision of 0.85, recall rate of 0.18, F1 score of 0.29, sensitivity of 0.18, specificity of 0.95 (Figure 3; Supplementary Table S1).

The clinical model combined with the clinical-laboratory signature and the demographic data including age, sex, ranked SBP, and ranked DBP. This model predicted moderate–severe WMLs with a determination R^2^ of 0.17, an AUC of 0.78, accuracy of 0.76, precision of 0.58, recall rate of 0.30, F1 score of 0.40, sensitivity of 0.30, specificity of 0.93 (Figure 3; Supplementary Table S1).

#### 3.3.3. The combined model for predicting the severity of WMLs

The combined model included age, sex, ranked SBP, ranked DBP, clinical-laboratory signature, the outputs of retina deep learning neural network based on the left and right retinal image (Table 3). The combined model could well recognize moderate–severe WMLs with an AUC of 0.95, accuracy of 0.90, precision of 0.85, recall rate of 0.79, F1 score of 0.82, sensitivity of 0.79, specificity of 0.95 (Figure 3; Supplementary Table S1).

**Table 3:** Multivariate logistics regression of combined and clinical models.

	Regression coefficient (M ± SD)	
	The combined model	The clinical model
Age	0.08 ± 0.01	0.089 ± 0.005
Sex/male	0.36 ± 0.36	0.027 ± 0.12
Ranked SBP	0.41 ± 0.32	0.39 ± 0.14
Ranked DBP	0.01 ± 0.25	0.28 ± 0.076
Clinical-laboratory signature	1.5 ± 0.59	4.35 ± 2.29
Left retina image	1.06 ± 0.27	/
Right retina image	5.59 ± 0.35	/

SBP, systolic blood pressure; DBP, diastolic blood pressure; M, mean; SD, standard deviation.

## 4. Discussion

To the best of our knowledge, this is the first study to use the combination of retina image, clinical features and laboratory data to develop a comprehensive model for predicting the severity of WMLs. The WMLs is a major threat to public health and early detection and routine follow-up are critical for controlling advance of WMLs and preventing deterioration of cognitive function. WMLs can be induced by various pathological mechanisms. Among all subtypes of WMLs, arteriosclerotic WMLs (namely, age- and vascular risk factor-related WMLs) are the most common in clinical practice (32). We excluded patients with autoimmune diseases, intoxication, metabolic brain disease, and hereditary WMLs. The patients with moderate–severe WMLs were significantly older and had higher SBP and DBP on admission. Previous studies have shown that advanced age and elevated blood pressure are significant causes of arteriosclerosis WMLs (11). Furthermore, elevated homocysteine, a well-known risk factor for arteriosclerosis (11), was found to be higher in the patients with moderate–severe WMLs, which is consistent with previous findings that homocysteine levels are positively correlated with the volume of arteriosclerotic WMLs (33–35). Hence, the WMLs investigated in the current study was further indicated to be of the arteriosclerotic subtype.

The WMLs significantly impair cognitive function (36) and are risk factors of ischemic stroke (37) and gait dysfunction (38). A convenient approach with high accuracy is helpful for screening and follow-up. In this study, we developed a comprehensive model that combined deep learning in retinal images and clinical laboratory data that can reliably predict the severity of WMLs.

ResNet has been applied to predict Alzheimer’s disease (39), diagnose myocardial infarction (40), analyze blood cells (41), diagnose COVID-19 (42), and diagnose malignant tumor (43) and tumor metastasis (44). In addition to the aforementioned research areas, ResNet deep learning models can also be used to detect retinal exudative lesions (45). The ResNet-18 is a robust deep learning network, where the convolutional layers are pre-trained and can start training the network without a large dataset. Therefore, this residual network overcomes the limitation of using deep learning for medical image studies with small datasets. Among all the models, the right retinal images and the combined model well predicted moderate–severe WMLs and outperformed the clinical model and clinical-laboratory signature. Furthermore, the deep learning network based on the right retinal images alone shared similar performance with the combined model in which the retina image was combined with clinical information. Our results were in line with the previous studies that reflect the pathophysiological basis that the retina shares similar vasculature and vascular risk factors (46–50). Previous studies have shown that chronic kidney disease (51) and dyslipidemia (11) were associated with WMLs. Similarly, in our study, the clinical-laboratory signature including certain laboratory indices of lipid metabolism (Apo-A1, TG) and kidney function (creatine) can predict the severity of WMLs. Thus, in our study, the abovementioned pathophysiological basis may explain the good performance of the comprehensive model combining deep learning neural network in retinal images with clinical laboratory data.

Traditionally, the retinal vascular lesion was manually evaluated according the presentations of exudates, vessel tortuosity, arteriovenous nicking and so on. However, this evaluation method was affected by raters’ bias and not quantitative. In our study, we focused on the entire retina image instead of the traditional evaluation method. We developed the deep learning model to analyze the entire image of the retina and show good AUC to predict WMLs. Our result further confirmed the findings of previous studies that the retinal lesions were associated with WMLs. In addition, the regression coefficient of retina deep learning model was higher than the traditional risk factors such as age and blood pressure in the combined model. This result indicated that the retinal images can predict the severity of WMLs.

Several limitations of this study should be mentioned. First, the WMLs severity was dichotomized as absent/mild or moderate–severe according to the Fazekas score in our study. Consequently, the models developed in our study could not quantitatively predict WMLs severity. Recent studies showed that severe WMLs were association with multiple neurological diseases such as cognitive deterioration (6, 52, 53) and ischemic stroke (37), while few study reported the association of mild WMLs and other neurological diseases. Thus, our model showed clinical significance in predicting moderate–severe WMLs. Second, our study was hospital-based and retrospectively designed. Some of retinal images were excluded due to the poor quality. The number of the eligible left retinal images included in our analysis was less than the right, which may explain that the inconformity of the performance of the deep learning neural network between left and right retinal images. Further studies may be needed to explore the potential difference of the predicting performance between left and right retinal images. On the other hand, the AUC of right retina image was high in our study, which indicates that the retinal image and deep learning network are capable of predicting the severity of WMLs.

## 5. Conclusion

In conclusion, we integrated demographic-clinical characteristics, clinical laboratory data and deep learning on retinal images to develop a comprehensive model. This comprehensive model accurately predicted the severity of WMLs and offered non-invasive, high-throughput and low-cost screening tool for early detection of moderate–severe WMLs in the population in community, resource-poor area or with contraindication of MRI.

## Data availability statement

The raw data supporting the conclusions of this article will be made available by the authors, without undue reservation.

## Ethics statement

The studies involving human participants were reviewed and approved by Ethics Committee of the First Affiliated Hospital of Sun Yat-sen University. Written informed consent for participation was not required for this study in accordance with the national legislation and the institutional requirements.

## Author contributions

LS and KZ carried out the study and wrote the manuscript with support from HH, WS, and JL. NC and WG develop the code for ResNet and statistics. HH conceived the original idea. HH and JR supervised the project. All authors contributed to the article and approved the submitted version.

## Funding

This work was supported by the Guangdong Key Laboratory of Non-human Primate Research (grant number 202OB121201006) and the Liuzhou Science and Technology Plan Project (grant number 2021YB0104B056).

## Conflict of interest

The authors declare that the research was conducted in the absence of any commercial or financial relationships that could be construed as a potential conflict of interest.

## Publisher’s note

All claims expressed in this article are solely those of the authors and do not necessarily represent those of their affiliated organizations, or those of the publisher, the editors and the reviewers. Any product that may be evaluated in this article, or claim that may be made by its manufacturer, is not guaranteed or endorsed by the publisher.

## Supplementary material

The Supplementary material for this article can be found online at: https://www.frontiersin.org/articles/10.3389/fneur.2023.1168836/full#supplementary-material

## Footnotes

^1^https://download.pytorch.org/models/resnet18-f37072fd.pth

^2^https://scikit-learn.org/
